# Supplementary material for: Phosphorylation induces sequence-specific conformational switches in the RNA polymerase II C-terminal domain
Source: Nat Commun. 2017 May 12;8:15233. doi: 10.1038/ncomms15233 (PMC5437310; doi:10.1038/ncomms15233)
Supplement: Supplementary Information — Supplementary Figures and Supplementary Tables [file ncomms15233-s1.pdf]

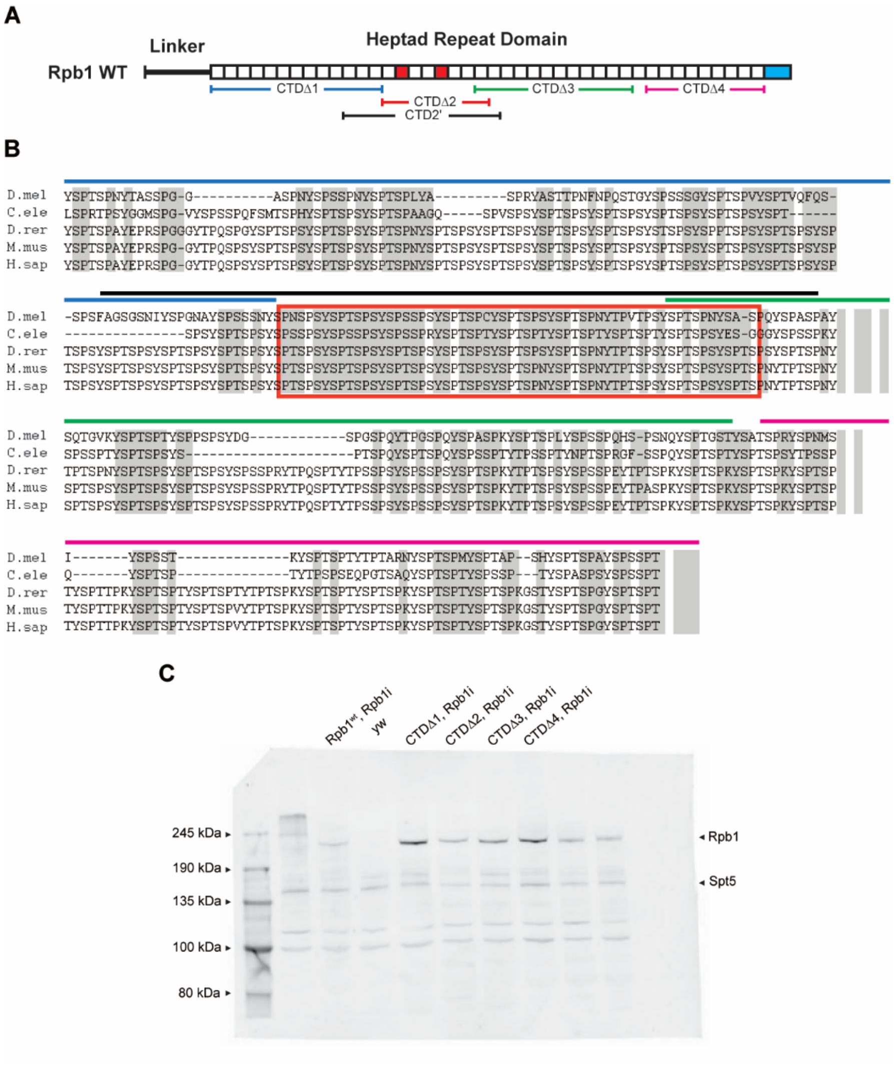

Supplementary Fig. 1. Identification of a region of the Rpb1 CTD that is essential for rescuing lethality caused by RNAi-mediated depletion of endogenous Rpb1 (a) Schematic representation of the C-terminal heptad repeat domain of Rpb1 showing the deleted regions from the respective transgenic strains and the recombinant CTD2′ construct. (b) Evolutionary conservation of the CTD across different species: Drosophila melanogaster (D. mel); Caenorhabditis elegans (C. ele); Danio rerio (D. rer); Mus musculus (M. mus); and Homo sapien (H. sap). Conserved amino acids are highlighted in gray. CTDΔ1, CTDΔ3, CTDΔ4 deletions are denoted by the blue, green and magenta bars, respectively. CTDΔ2 deletion (red box) contains a highly conserved region. The recombinant CTDΔ2′ construct is denoted by the black line. (c) Uncropped image showing the western blot analysis of the expression of Rpb1 derivatives from Fig. 1c. The lanes shown in Fig. 1c are labeled for clarity.

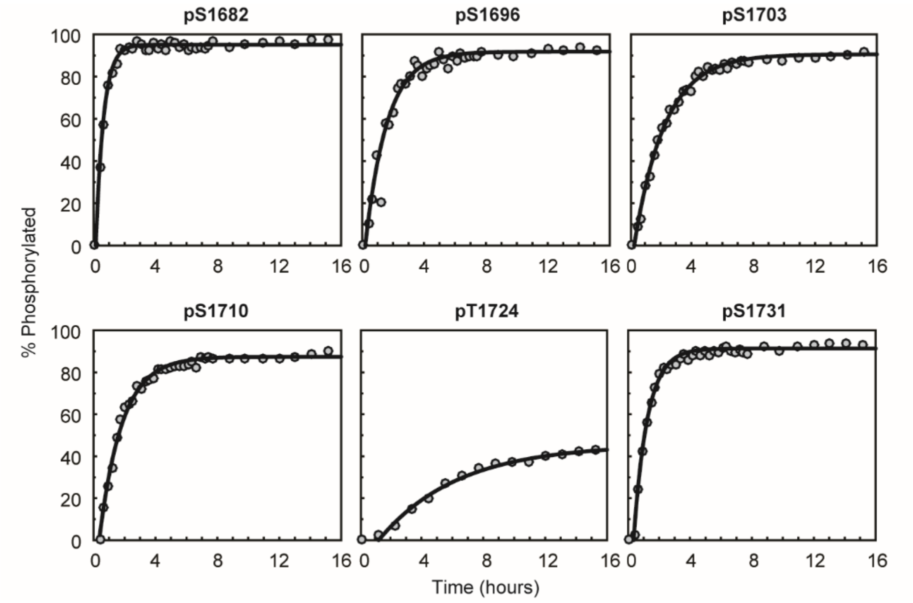

Supplementary Fig. 2. Kinetics of DmP-TEFb phosphorylation of CTD2′ measured by RT-NMR. Peak intensities for unphosphorylated and phosphorylated species were extracted from 2D $^{1}H,^{15}N$ correlation spectra and the percentage of phosphorylation was plotted as a function of time. Fits were performed as described in the online methods.

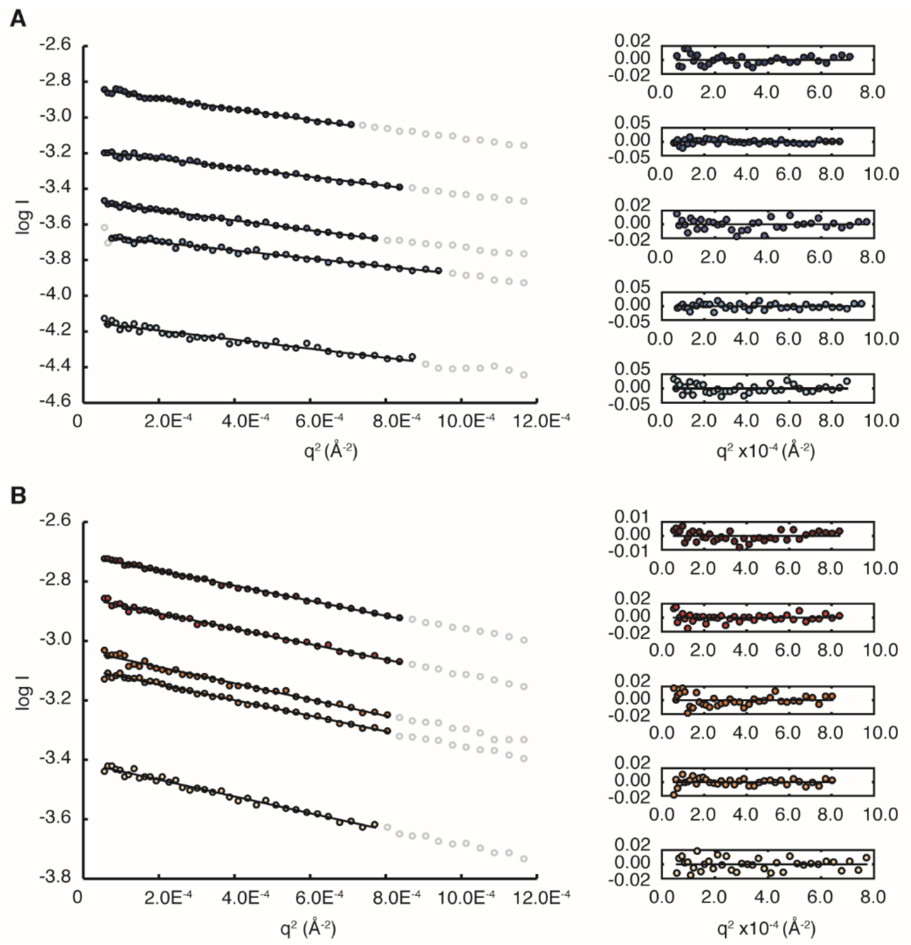

Supplementary Fig. 3. Small angle X-ray scattering reveals no significant changes in R_g for CTD2´upon serine 5 phosphorylation. (A) Guinier fits for unphosphorylated CTD2´ collected at protein concentrations of 4.0, 5.9, 7.0, 8.8, and 11.4 mg/ml (left) with residuals (right). (B) Guinier fits for hyper-pSer5 CTD2´ collected at protein concentrations of 4.1, 6.8, 7.2, 8.7, 10.0 mg/ml (left) with residuals (right). Data over the range of $qR_g < 0.8$ was fit to the Guinier approximation in MATLAB using the method of non-linear least squares.

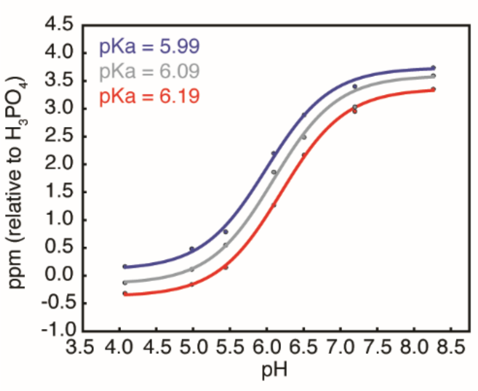

Supplementary Fig. 4. Phosphoserine pKa values determined by ^{31}P NMR spectroscopy. pKa values for hyper-pSer5 CTD2′ were determined by non-linear least squares fitting in MATLAB. The chemical shifts of well resolved phosphoserine resonances (δ) were plotted as a function of pH and fit to $\delta = [\delta^{2-} (10^{pH-pKa}) + \delta^{-}] / [1 + 10^{pH-pKa}]$, where δ^{-} and δ^{2-} are the chemical shifts of the mono-anionic and di-anionic phosphate species, respectively. Data points and best fit lines are shown for three phosphoserine resonances, representing fitted pKa values at the acidic (blue) and basic (red) ends of the range, as well as a representative intermediate fitted value (gray).

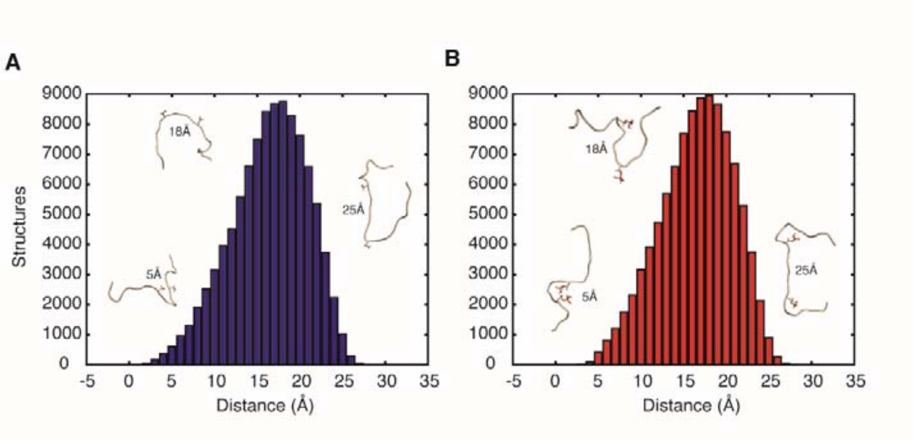

Supplementary Fig. 5. Random coil conformations of CTD2´ heptads separate consecutive Ser5s or pSer5s by distances that exceed the Debye length. (a) Histogram showing the distribution of inter-Ser5-Ser5 distances computed from 100,000 random coil structures of YSPTSPSYSPTSSPSYSPTSPCYSPTSPS generated using traDES. (b) Histogram showing the distribution of inter-pSer5-pSer5 distances computed from 100,000 random coil structures of YSPTpSPSYSPTSpSPSYSPTpSPCYSPTpSPS generated using traDES. Distances were calculated from Oγ atoms in Ser5/pSer5 residues located within the two central heptads of each construct. Three representative structures are shown as insets to each panel.

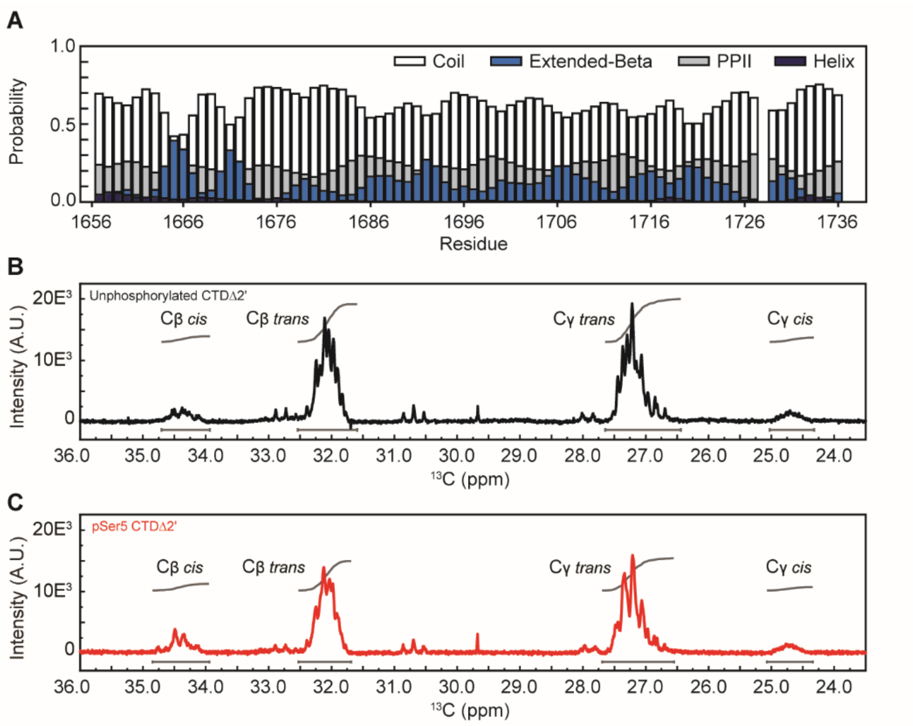

Supplementary Fig. 6. Structural characterization of unphosphorylated CTD2´ by NMR spectroscopy. (a) Secondary structure populations for unphosphorylated CTDΔ2′ determined with Δ2D using NMR chemical shifts (b) Proline Cβ and Cγ chemical shifts from the ^{13}C spectrum of unphosphorylated CTDΔ2′. These peaks represent the population of *cis* and *trans* isomers averaged over all proline residues (95% *trans*, 5% *cis*) (c) In the hyper-pSer5 state, the population of *cis*-proline is enriched ~2-fold.

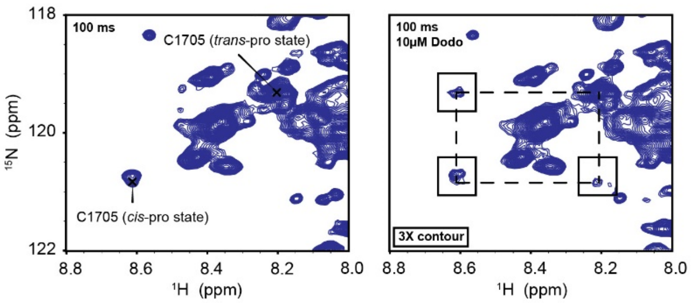

Supplementary Fig. 7. Proline *cis-trans* isomerization in Hyper-pSer5 CTD2´ probed by NMR spectroscopy. 2D H-N correlation spectra for the measurement of ^{15}N ZZ-exchange collected on 1 mM hyper-pSer5 CTDΔ2′, in the absence of Dodo, at a relaxation delay of 100 ms (left). In the presence of 10 μM Dodo, ZZ exchange pairs can be observed. (right)

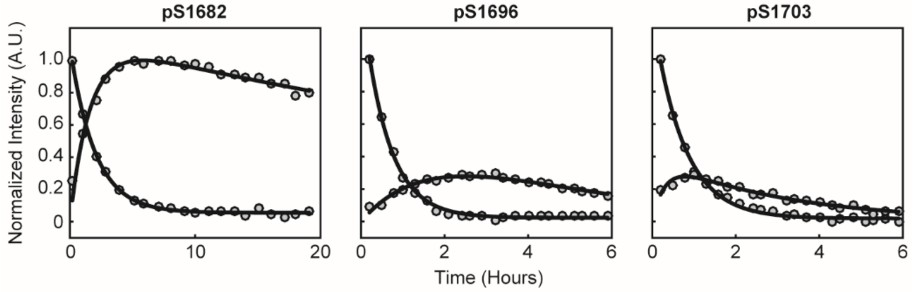

Supplementary Fig. 8. Kinetics of Ssu72-Symplekin dephosphorylation of Hyper-pSer5 CTD2´ measured by RT-NMR. Extracted peak intensities from 2D ^{1}H,^{15}N correlation spectra were plotted as a function of time and fit as described in methods.

Supplementary Table 1. Results of the rescue assay.

Transgenic Strain	curly wing females	curly wing males	straight wing females	straight wing males
yw	99	49	68	62
Rpb1^{wt}, Rpb1i	53	18	37	67
CTDΔ1, Rpb1i	38	15	9	3
CTDΔ2, Rpb1i	39	27	0	0
CTDΔ3, Rpb1i	25	27	7	10
CTDΔ4, Rpb1i	36	34	13	34

Numbers of progeny with particular phenotypes and the percentages of straight-winged progeny are calculated as shown in (Fig. 1d). Rpb1i corresponds to the Gal4-activated, UAS-Rpb1i transgene. Rpb1WT and CTDΔ1 to Δ4 correspond to the Gal4-activated UAS-Rpb1 transgenes.

Supplementary Table 2. Kinetic parameters for DmP-TEFb phosphorylation of CTD2′.

Residue:	Sequence	k_{app} (hour^{-1})
S1675	YSPSSSN	0.15 ± 0.03
S1682	YSPNSPS	1.39 ± 0.20
S1689	YSPTSPS	0.78 ± 0.09
S1696	YSPSSPS	0.71 ± 0.06
S1703	YSPTSPC	0.76 ± 0.09
S1710	YSPTSPS	0.68 ± 0.05
S1717	YSPTSPN	0.78 ± 0.11
T1724	YTPVTPS	0.26 ± 0.02
S1731	YSPTSPN	0.95 ± 0.11
S1737	YSASPQ	0.09 ± 0.08

Supplementary Table 3. SAXS data collection and scattering derived parameters for unphosphorylated and hyper-pSer5 CTD2′.

Data Collection Parameters:	CTD2′	pSer5 CTD2′
Instrument	CHESS G1 Station	CHESS G1 Station
Beam diameter:	250 μm × 250 μm	250 μm × 250 μm
Wavelength (Å)	1.244	1.244
E (keV)	9.963	9.963
qRange (Å^{-1})	0.007- 0.7	0.007- 0.7
Flux (photons/s)	8 x 10ˆ11 @ 51 ma	8 x 10ˆ11 @ 51 ma
Exposure Time (s)	60	60

Concentration (mg ml^{-1})	4-11	4-11
Temperature (K)	296	296
**Structural Parameters:**		
†*R*g (Å) (Guinier)	28.01 ± 0.69	28.27 ± 0.26
*R*g (Å) [P(r)]	27.11 ± 0.25	27.52 ± 0.84
D_{max} (Å)	111.13‡	112.48*
**Software Used:**		
Primary data reduction	BioXtasRAW	BioXtasRAW
Guinier fitting	MATLAB	MATLAB
P(r) calculations	PRIMUSqt/GNOM	PRIMUSqt/GNOM

† Reported as the average ± S.E.M., ‡ Dmax shown for 4.0 mg/ml sample, * Dmax shown for 10.0 mg/ml sample.

Supplementary Table 4. Kinetic parameters for Ssu72-Symplekin dephosphorylation of Hyper-pSer5 CTD2′.

**Residue:**	**Sequence**	**k_{app} (hour^{-1})**
pS1675	YSPSpSSN	N.D.
pS1682	YSPNpSPS	0.02 ± 0.01
pS1689	YSPTpSPS	0.39 ± 0.05
pS1696	YSPSpSPS	0.40 ± 0.06
pS1703	YSPTpSPC	0.33 ± 0.05
pS1710	YSPTpSPS	0.44 ± 0.06
pS1717	YSPTpSPN	1.10 ± 0.07
pT1724	YTPVpTPS	N.D.
pS1731	YSPTpSPN	1.19 ± 0.05
pS1737	YSApSPQ	N.D.
